# Life-style and metabolic factors do not affect risk for glioma: a prospective population-based study (The Cohort of Norway)

**DOI:** 10.3389/fonc.2024.1471733

**Published:** 2024-12-04

**Authors:** Anamaria Gheorghiu, Cathrine Brunborg, Tom B. Johannesen, Eirik Helseth, John-Anker Zwart, Markus K. H. Wiedmann

**Affiliations:** ^1^ Department of Neurosurgery, Bagdasar-Arseni University Hospital, Bucharest, Romania; ^2^ Faculty of Medicine, University of Oslo, Oslo, Norway; ^3^ Centre for Biostatistics and Epidemiology, Research Support Services, Oslo University Hospital, Oslo, Norway; ^4^ The Cancer Registry of Norway, Oslo, Norway; ^5^ Department of Neurosurgery, Oslo University Hospital, Oslo, Norway; ^6^ Department of Research and Innovation, Division of Clinical Neuroscience, Oslo University Hospital, Oslo, Norway

**Keywords:** glioma, glioblastoma, metabolic syndrome, diabetes, obesity, brain tumor, risk factor, smoking

## Abstract

**Background:**

The identification of modifiable risk factors for intracranial glioma remains a significant challenge. While lifestyle factors and metabolic syndrome are well-established risk factors for various other cancers, their association with glioma risk remains unclear.

**Objectives:**

This study aims to conduct a comprehensive analysis of lifestyle factors and metabolic factors in relation to glioma risk.

**Methods:**

The Cohort of Norway (CONOR) is a prospective, population-based health survey encompassing anthropometric measurements, blood tests and health questionnaires. CONOR data were linked to the National Cancer Registry to identify incident glioma cases. Follow-up time was calculated in person-years from the baseline examination until the date of glioma diagnosis, death, or the end of the follow-up period. Cox proportional hazards regression was used to calculate hazard ratios (HR).

**Results:**

The study cohort included 160,938 women and men. Over 2.8 million person-years of follow-up, 319 intracranial gliomas were diagnosed. Lifestyle factors such as physical activity, alcohol consumption, smoking, and marital status were not associated with glioma risk. There was no increased glioma risk among participants with diabetes mellitus or hypertension. Furthermore, metabolic syndrome in both women and men was not associated with an elevated risk of glioma. Blood lipids, including total cholesterol, triglycerides, and HDL, were not linked to glioma risk. However, increasing LDL levels were associated with a decreased risk of glioma in men (HR per category 0.84; 95% CI 0.74-0.96), but not in women.

**Conclusion:**

This is the first comprehensive prospective cohort study to evaluate potentially modifiable risk factors for glioma. Our findings do not support previously suggested associations between smoking, alcohol consumption, or metabolic syndrome and glioma risk.

## Introduction

Glioma is the most common primary malignant brain tumor, with an incidence rate of 4.5 per 100,000 person-years (1). Glioblastoma, the most aggressive subtype, constitutes 59% of all gliomas (1). Despite advancements in treatment, gliomas generally have a poor prognosis, underscoring the critical need for effective prevention strategies. Identifying modifiable risk factors for glioma is essential for developing preventive measures.

Recent studies have highlighted the potential role of metabolic syndrome as a risk factor for various cancers, including colorectal, pancreatic, postmenopausal breast, and bladder cancers (2–7).

Metabolic syndrome encompasses a cluster of conditions such as glucose intolerance, hypertension, dyslipidemia, and obesity. Notably, glucose intolerance alone has been implicated as a significant risk factor for several cancers, including hepatocellular, hepatobiliary, pancreatic, breast, ovarian, endometrial, and gastrointestinal cancers (7–11).

Modifiable life-style factors, such as body mass, physical activity, alcohol consumption and smoking may further amplify increased cancer risk in patients with glucose intolerance. Conversely, allergic conditions have been reported to decrease glioma risk by approximately 30%, though the underlying mechanisms remain poorly understood (12–15). To date, no study has comprehensively integrated these factors to assess their combined impact on glioma risk.

This study aims to evaluate the association between lifestyle and metabolic factors, and glioma risk in a large, prospective Norwegian cohort of adult women and men. By leveraging data from the Cohort of Norway (CONOR), which includes extensive health and lifestyle information, we want to provide a more comprehensive understanding of the potentially modifiable risk factors for glioma.

## Materials and methods

### Study population

The Cohort of Norway (CONOR) is a comprehensive health survey designed to reflect the population of Norway in terms of exposure distribution and health status. This includes anthropometric measurements, blood tests, health questionnaires, lifestyle factors, and socio-economic variables. Contributing regional health surveys harmonized approximately 50 core CONOR questions, with the inaugural survey conducted in Tromsø in 1994 (16). Detailed information about the study is available on the CONOR website (https://www.fhi.no/en/studies/conor/about-conor—data-from-several-regional-health-studies/).

For all CONOR surveys, invitation letters were dispatched two weeks prior to the scheduled appointments, containing a questionnaire and detailed information about the study. During the screening appointments, participants underwent a physical examination and blood samples were collected. Additionally, supplementary questionnaires were provided to the participants for completion and return by mail (16).

Body weight and height were measured according to a standardized protocol. Heart rate, as well as systolic and diastolic blood pressures, were measured using an automatic device following a 2-minute period of seated rest, with three recordings taken at 1-minute intervals.

A total of 309,742 individuals were invited to participate in the CONOR survey between 1994 and 2003, with an overall participation rate of 56%.

### Linkage of databases

Norwegian residents have a unique 11-digit identification number universally utilized for personal identification. This identifier facilitated the linkage of CONOR participants to the Norwegian Cancer Registry, enabling the identification of any tumor diagnoses during the follow-up period. Additionally, linkage to the Norwegian Tax Administration permitted the determination of the status of all study participants (i.e., resident, emigrated, deceased) as of the last follow-up date (December 15, 2018). The Norwegian Cancer Registry has operated as a national cancer registry with mandatory reporting by clinicians and pathology departments since 1952.

### Outcome characteristics

Based on the International Classification of Diseases for Oncology, Third Edition (ICD-O-3), glioma was defined using the morphology codes 9380-9384 and 9391-9460. To specify the intracranial location, these morphology codes were combined with the topography codes 193.0–193.2 and 195.3–195.5 from the International Classification of Diseases, Seventh Revision (ICD-7).

### Categorization of independent variables

#### Civil status, education, physical activity, smoking, alcohol consumption and allergy

Civil status was categorized as married or partner, single, widowed, and separated. Educational attainment was categorized into 0-10 years, 11-15 years, and over 15 years. Physical activity levels were assessed based on survey questions regarding the extent of regular physical activity per week. This was defined by the total time of activity per week in hours (none, <1, 1-2, or ≥3) and the intensity of activity (light activity: not sweating or out of breath, and hard activity: sweating or out of breath). Activity levels were classified as follows: None: No physical activity. Low activity level: 1-2 hours of light activity per week, with no hard activity. Moderate activity level: Less than 1 hour of hard activity per week, with or without 1-2 hours of light activity, or at least 3 hours of light activity alone. High activity level: At least 1-2 hours of hard activity per week, or up to 1 hour of hard activity combined with a minimum of 2 hours of light activity. Smoking status was categorized into current daily smokers, former smokers, and never smokers. Alcohol consumption was categorized as once per month or less, 2-3 times per month, once per week, or several times per week. An allergic condition was defined as having hay fever, asthma, or using allergy medication for ≥1 month per year.

#### Anthropometric measures, blood pressure, blood lipids, diabetes mellitus and definition of metabolic syndrome

The height and weight of study participants were measured by trained personnel at baseline. Body mass index (BMI) was calculated as weight divided by height squared (kg/m²) and categorized into four groups: <20, 20-24.9, 25-29.9, and ≥30 kg/m², as well as per 5 kg/m² increase in BMI. Overweight was defined as a BMI of 25-29.9 kg/m², obesity as a BMI of ≥30 kg/m², underweight as a BMI of <20 kg/m², and a BMI of 20-24.9 kg/m² was used as the reference category (17).

Hypertension was defined as a mean systolic blood pressure (BP) of ≥140 mmHg or a diastolic BP of ≥90 mmHg, or the use of antihypertensive medication. The continuous variables systolic and diastolic BP, as well as blood lipids (total cholesterol, triglycerides, HDL, and LDL), were divided into quartiles. Diabetes mellitus was defined as either receiving treatment for diabetes or having glucose intolerance, indicated by a fasting glucose level of ≥5.6 mmol/L. LDL cholesterol was calculated using the Friedewald formula: LDL = Total Cholesterol - (HDL + (Triglycerides mmol/L)/2.2). Metabolic syndrome was defined based on the Adult Treatment Panel III (ATP III) criteria, which requires the presence of at least three of the following five traits (18): 1.) Abdominal obesity, defined as a BMI of ≥30 kg/m² (instead of waist circumference); 2.) Serum triglycerides of ≥1.7 mmol/L or receiving drug treatment for elevated triglycerides; 3.) Serum HDL cholesterol of <1.3 mmol/L for women and <1.0 mmol/L for men; 4.) BP of ≥130/85 mmHg or receiving drug treatment for elevated blood pressure; 5.) Fasting plasma glucose (FPG) of ≥5.6 mmol/L or receiving drug treatment for elevated blood glucose.

### Definition of follow-up time

Follow-up time was calculated in person-years from the date of the baseline examination (which included measurements of BMI, blood pressure, and blood sampling) until the earliest of the following events: glioma diagnosis, any other cancer diagnosis, emigration, death from any cause, or the end of follow-up on December 15, 2018.

### Statistical analysis

Cox proportional hazards regression, using attained age at study baseline as the time axis, was performed to calculate hazard ratios (HR) with 95% confidence intervals (CI).

Risk factors for glioma included in the multivariable analyses were selected based on prior knowledge from the literature, pre-defined ATP III criteria for metabolic factors, and identification by univariable analysis, with careful consideration of multi-collinearity. Multi-collinearity between risk factors was assessed using Spearman’s correlation coefficient with a cut-off of ≥0.7.

The proportional hazards assumptions were tested by plotting the logarithm of the integrated hazards (log-log survival plots) and by conducting Schoenfeld tests. A two-sided probability with a significance level of 0.05 was used throughout. Statistical analyses were performed using STATA/SE statistical software, version 17/18 (StataCorp, College Station, TX, USA).

### Ethical statement

This study was approved by the Regional Committee for Ethics in Medical Research (REK No 2011/428).

## Results

### Characteristics of the study population

The study cohort consisted of 160,938 individuals (women and men) aged between 20 and 80 years, with women comprising 51% of the population. At the time of study inclusion, 25% of participants were younger than 40 years. The median follow-up time was 19.4 years (IQR 16.3-22.2 years). Over a total of 2,877,646 person-years of follow-up, 319 intracranial gliomas were diagnosed histopathologically. Characteristics of the study cohort are detailed in **Table 1**.

**Table 1:** Baseline characteristics of the population at risk and intracranial glioma cases in the Cohort of Norway (CONOR).

	Population at risk	Glioma
No. of participants	160 938	319
Mean age at study baseline	48.9 (SD 14.5)	50.3 (SD 12.3)
BMI (kg/m^2^)		
<20	6028	13
20-24.9	65184	117
25-29.9	65928	146
≥30	23798	43
Mean BMI (kg/m^2^)	26 (SD 4.1)	26.2 (SD 4.0)
Education (≥15 years) (%)	16	13
Married (%)	61.3	65.2
Hypertension (%)	36.5	39.8
Diabetes (%)	4.3	3.1
Metabolic syndrome (%)	13.9	10.9
LDL >4.1 mmol/L or statin use (%)	33.0	32.0
Hard physical activity ≥ 2 times per week	36.8	37.6
Allergic condition	18.9	17,9
Current smoker (%)	30.9	26.9
Alcohol consumption ≥ 2 times per week	11.9	15.0

SD standard deviation; No number; BMI body mass index; BP blood pressure; LDL low density lipoprotein.

### Socio-economic and life-style factors

**Figure 1:**
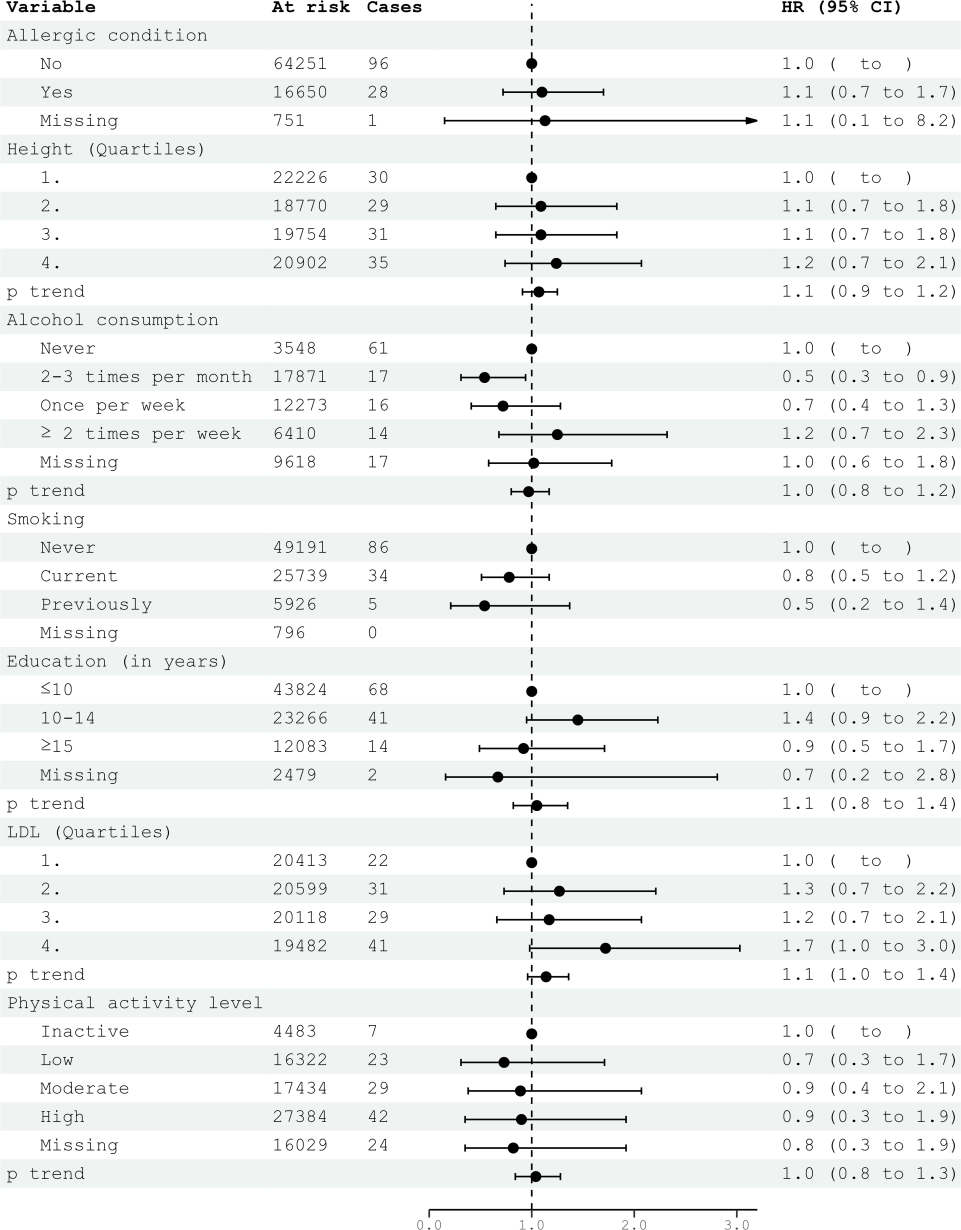
HRs (95% CIs) in multivariable models and risk for glioma among women in CONOR. Cox regression models with age as the time axis, including allergic condition, height, alcohol consumption, smoking, education, LDL and physical activity in a multivariable analysis. HR, hazard ratios; CI, confidence intervals; LDL, Low Density Lipoprotein.

**Figure 2:**
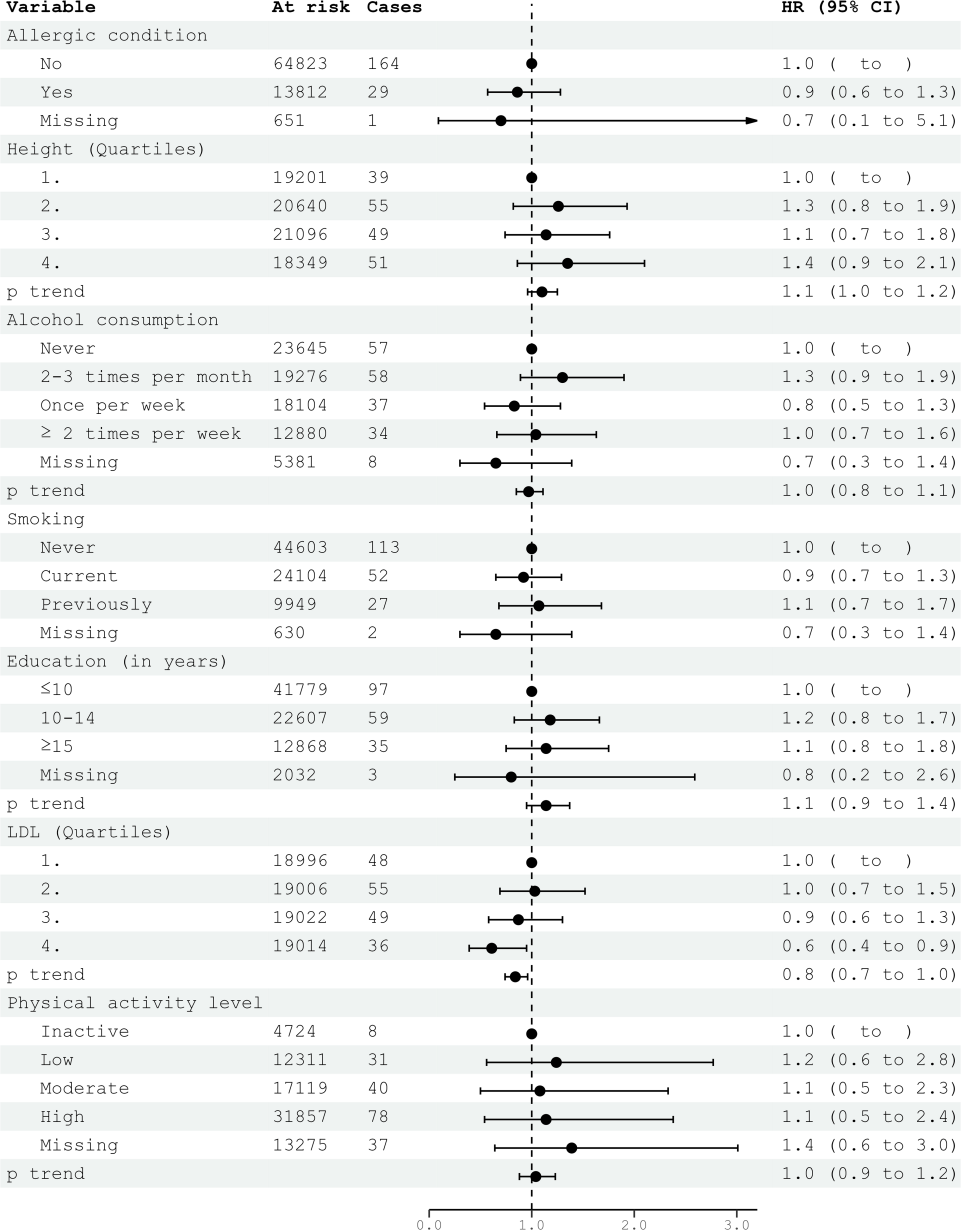
HRs (95% CIs) in multivariable models and risk for glioma among men in CONOR. Cox regression models with age as the time axis, including allergic condition, height, alcohol consumption, smoking, education, LDL and physical activity in a multivariable analysis. HR, hazard ratios; CI, confidence intervals; LDL, Low Density Lipoprotein.

Civil status was not associated with glioma risk. In univariable analysis, an education level of 10-15 years was associated with an increased risk of glioma (HR 1.32; 95% CI 1.03-1.70) (**Table 2**). However, this association did not persist in multivariable models for both women and men (**Figures 1**, **2**). Neither cigarette smoking, alcohol consumption, nor the level of physical activity were associated with glioma risk (**Table 2**).

**Table 2:** HRs (95% CIs) for life-style factors and risk for glioma among women and men in CONOR (univariable analyses).

	No. at risk	Cases	HR (95% CI)
Civil status			
Single	36471	56	Ref
Married	98722	208	0.98 (0.71-1.36)
Widow	8888	12	0.81 (0.41-1.59)
Separated	16241	42	1.24 (0.81-1.90)
Missing	616	1	0.67 (0.09-4.90)
Education (yrs)			
≦10	85603	165	Ref
10 to 14	45873	100	**1.32 (1.03-1.70)**
≧ 15	24951	49	1.15 (0.84-1.60)
Missing	4511	5	0.68 (0.28-1.67)
p trend			1.12 (0.97-1.30)
Cigarette smoking			
None	93794	199	Ref
Current	49843	86	0.83 (0.65-1.08)
Previous	15875	32	1.01 (0.69-1.47)
Missing	1426	2	0.69 (0.17-2.80)
Physical activity level			
Inactive	9207	15	Ref
Low	28633	54	1.09 (0.61-1.94)
Moderate	34553	69	1.14 (0.65-1.99)
High	59241	120	1.18 (0.69-2.02)
Missing	29304	61	1.16 (0.66-2.04)
p trend			1.05 (0.92-1.19)
Regular alcohol consumption			
≦1 times per month	59125	118	Ref
2-3 times per month	37147	75	0.94 (0.70-1.27)
Once per week	30377	53	0.76 (0.55-1.06)
≧ 2 times per week	19290	48	1.06 (0.75-1.49)
Missing	14999	25	0.80 (0.52-1.23)
P trend			0.98 (0.87-1.09)

Cox regression models with age as the time axis including sex as confounding variable. Univariable analysis. HRs, hazard ratios; CIs, confidence intervals; Ref, reference; yrs years.

### Anthropometric measures, blood pressure and blood lipids

Overweight or obesity, as measured by body mass index (BMI), were not associated with glioma risk in either women or men (**Tables 3**, **4**). However, in underweight men, there was a positive association with glioma risk (HR 3.08; 95% CI 1.48-6.44) (**Table 3**). Body height was not significantly associated with glioma risk in either women or men (**Tables 3**, **4**).

**Table 3:** HRs (95% CIs) for anthropometric data, blood pressure and blood lipids and risk for glioma among women in CONOR (univariable analyses).

	No. at risk	Cases	HR (95% CI)
Height in quartiles			
1.	22226	30	Ref
2.	18770	29	1.07 (0.64-1.79)
3.	19754	31	1.10 (0.66-1.84)
4.	19754	35	1.24 (0.75-2.05)
*P trend*			1.07 (0.91-1.25)
BMI category (kg/m^2^)			
<20	4649	5	0.82 (0.33-2.05)
20-24.9	37181	55	Ref
25-29.9	27054	44	1.04 (0.70-1.56)
≥30	12768	21	1.08 (0.65-1.79)
*p trend*			0.95 (0.81-1.12)
Systolic BP (quartiles)			
1.	20940	29	Ref
2.	21111	35	1.08 (0.66-1.78)
3.	20804	33	0.93 (0.56-1.56)
4.	18797	28	0.90 (0.51-1.59)
*p trend*			0.95 (0.80-1.14)
Diastolic BP (quartiles)			
1.	21567	26	Ref
2.	20823	35	1.26 (0.76-2.11)
3.	19951	31	1.07 (0.63-1.82)
4.	19311	33	1.12 (0.66-1.91)
*P trend*			1.01 (0.86-1.19)
Cholesterol (quartiles)			
1.	21622	28	Ref
2.	20394	32	1.05 (0.63-1.76)
3.	19939	24	0.75 (0.43-1.32)
4.	19697	41	1.27 (0.75-2.16)
*p trend*			1.05 (0.88-1.25)
Triglycerides (quartiles)			
1.	21454	33	Ref
2.	20825	32	0.93 (0.57-1.53)
3.	19950	33	0.99 (0.61-1.62)
4.	19423	27	0.83 (0.49-1.40)
*p trend*			0.95 (0.81-1.12)
HDL (quartiles)			
1.	20925	25	Ref
2.	23829	47	1.59 (0.98-2.59)
3.	16985	24	1.13 (0.64-1.98)
4.	19913	29	1.11 (0.65-1.90)
*p trend*			0.98 (0.84-1.15)
LDL (quartiles)			
1.	20413	22	Ref
2.	20599	31	1.26 (0.72-2.18)
3.	20118	29	1.13 (0.64-2.00)
4.	19482	41	1.61 (0.92-2.81)
*p trend*			1.15 (0.96-1.37)

Cox regression models with age as the time axis. HRs, hazard ratios; CIs, confidence intervals; Ref, reference; BMI, body mass index; BP, blood pressure; HDL, high density lipoprotein; LDL, low density lipoprotein.

**Table 4:** HRs (95% CIs) for anthropometric data, blood pressure and blood lipids and risk for glioma among men in CONOR (univariable analyses).

	No. at risk	Cases	HR (95% CI)
Height in quartiles			
1.	19,201	39	Ref
2.	20,640	55	1.28 (0.84-1.93)
3.	21,096	49	1.14 (0.75-1.75)
4.	18,349	51	1.45 (0.94-2.22)
*P trend*			1.10 (0.96-1.25)
BMI category (kg/m^2^)			
<20	1,379	8	3.08 (1.48-6.44)
20-24.9	28,003	62	Ref
25-29.9	38,874	102	1.10 (0.80-1.52)
≥30	11,030	22	0.86 (0.53-1.40)
*p trend*			0.88 (0.71-1.07)
Systolic BP (quartiles)			
1.	20,421	49	Ref
2.	19,486	51	1.05 (0.71-1.56)
3.	19,623	47	0.93 (0.62-1.39)
4.	19,756	47	0.88 (0.58-1.34)
*p trend*			0.95 (0.83-1.08)
Diastolic BP (quartiles)			
1.	20,501	43	Ref
2.	20,152	45	0.93 (0.61-1.42)
3.	19,144	56	1.13 (0.75-1.70)
4.	19,489	50	0.95 (0.62-1.45)
*P trend*			1.00 (0.88-1.14)
Cholesterol (quartiles)			
1.	19,836	50	Ref
2.	19,938	57	0.98 (0.67-1.44)
3.	19,804	49	0.80 (0.53-1.19)
4.	19,708	38	0.59 (0.38-0.91)
*p trend*			0.84 (0.73-0.95)
Triglycerides (quartiles)			
1.	19,587	49	Ref
2.	19,929	51	0.98 (0.66-1.45)
3.	19,959	52	0.99 (0.67-1.47)
4.	19,811	42	0.80 (0.53-1.22)
*p trend*			0.94 (0.82-1.06)
HDL (quartiles)			
1.	19,829	43	Ref
2.	20,629	55	1.21 (0.81-.180)
3.	19,036	40	0.94 (0.61-1.44)
4.	19,792	56	1.21 (0.81-1.81)
*p trend*			1.03 (0.91-1.17)
LDL (quartiles)			
1.	18,996	48	Ref
2.	19,006	55	1.01 (0.68-1.49)
3.	19,022	49	0.85 (0.57-1.27)
4.	19,014	36	0.59 (0.38-0.92)
*p trend*			0.85 (0.74-0.97)

Cox regression models with age as the time axis. HRs, hazard ratios; CIs, confidence intervals; Ref, reference; BMI, body mass index; BP, blood pressure; HDL, high density lipoprotein; LDL low density lipoprotein.

Systolic and diastolic blood pressure measurements were not associated with glioma risk in women or men (**Tables 3**, **4**). Blood lipids, including total cholesterol, triglycerides, and HDL, were not associated with glioma risk in women or men overall. However, LDL cholesterol, as calculated by the Friedewald formula, was inversely related to glioma risk in men (HR per category 0.84; 95% CI 0.74-0.96) (**Table 4**, **Figure 2**), but not in women (**Table 3**, **Figure 1**).

### Metabolic factors and metabolic syndrome

**Figure 3:**
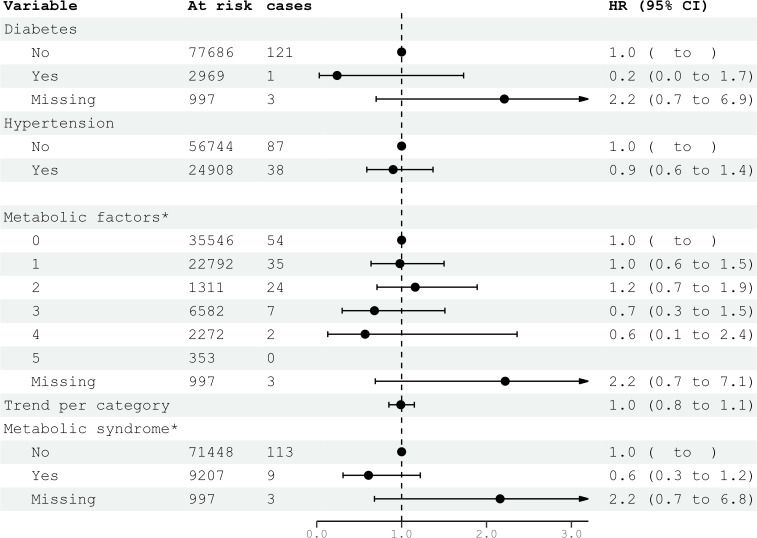
HRs (95% CIs) for metabolic factors and metabolic syndrome and risk for glioma among women in CONOR. Multivariable Cox regression model including all five metabolic factors (obesity, HT, increased serum triglycerides, decreased HDL, glucose intolerance or diabetes mellitus) and education. Correlation matrix of coefficients of cox model did not confirm multi-collinearity between variables. HR, hazard ratios; CI, confidence intervals.

**Figure 4:**
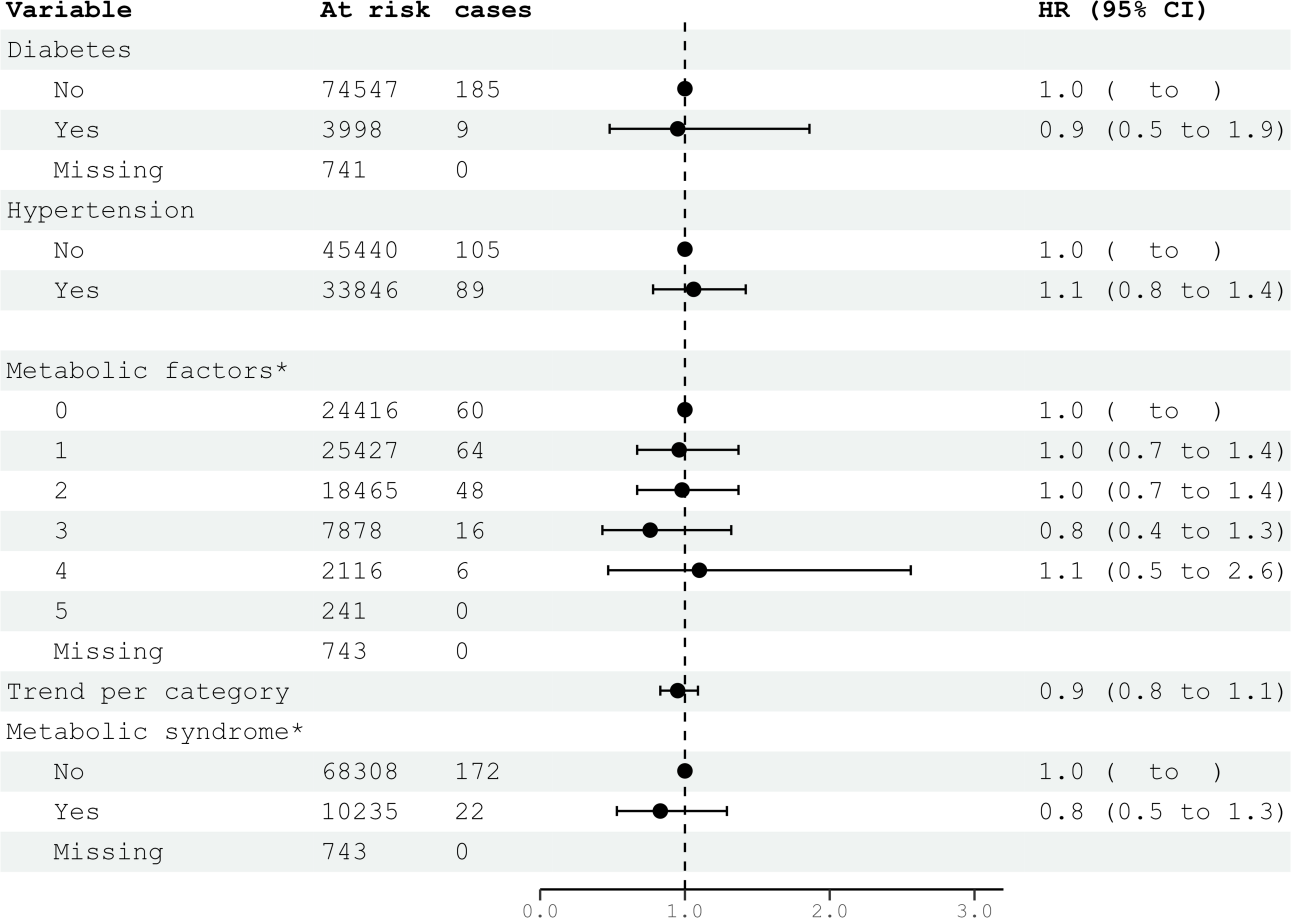
HRs (95% CIs) for metabolic factors and metabolic syndrome and risk for glioma among men in CONOR. ^*^Multivariable Cox regression model including all five metabolic factors (obesity, HT, increased serum triglycerides, decreased HDL, glucose intolerance or diabetes mellitus) and education. Correlation matrix of coefficients of cox model did not confirm multi-collinearity between variables. HR, hazard ratios; CI, confidence intervals.

Diabetes mellitus, glucose intolerance, and hypertension (BP ≥140/90 mmHg) were not associated with glioma risk in either women or men (**Figures 3**, **4**). Metabolic factors, as defined by the ATP III criteria, with BMI ≥30 kg/m² as the proxy for abdominal obesity, were also not associated with glioma risk. Additionally, metabolic syndrome, defined by the presence of at least three out of five metabolic factors, was not associated with an increased risk of glioma in either women or men (**Figures 3**, **4**).

### Allergic conditions

Allergic conditions, defined as asthma, hay fever, or the use of anti-allergic medication for ≥1 month per year, were not associated with glioma risk. This lack of association was consistent for both hay fever and asthma when analyzed separately (**Table 5**).

**Table 5:** HRs (95% CIs) for allergic condition, hay fever and asthma and risk for glioma among women and men in CONOR (univariable analyses).

	No. at risk	Cases	HR (95% CI)
Allergic condition*			
No	129,074	260	Ref
Yes	30,462	57	0.98 (0.73-1.31)
Missing	1,402	2	0.80 (0.20-3.23)
Hay fever			
No	106,230	206	Ref
Yes	20,595	40	1.06 (0.75-1.49)
Missing	34,113	73	1.13 (0.86-1.49)
Asthma			
No	146,090	292	Ref
Yes	13,097	24	0.98 (0.65-1.49)
Missing	13,097	3	0.99 (0.32-3.09)

Cox regression models with age as the time axis, stratified for sex. *Either hay fever, asthma or using allergy medicine ≥ 1 month per year. HRs, hazard ratios; CIs, confidence intervals; Ref, reference.

### Multivariable analyses

In the multivariable analyses, we included variables that had been previously shown to be associated with glioma risk in other studies or were suspected candidates based on univariable analysis. Additionally, metabolic syndrome and metabolic factors constituted a separate multivariable model. The results of the multivariable analysis stratified by sex are presented in **Figures 1**, **2**. Increasing levels of LDL were inversely associated with glioma risk in men (HR per category 0.84; 95% CI 0.74-0.96), but not in women (HR per category 1.14; 95% CI 0.96-1.36). The previously observed association with education was attenuated in the multivariable analyses when stratified by sex. Metabolic syndrome was not associated with glioma risk in either women or men in the multivariable analyses (**Figures 3**, **4**).

## Discussion

In this large prospective cohort study of women and men, the risk of glioma was generally not associated with metabolic risk factors. There was also no significant association between lifestyle factors such as smoking, alcohol consumption, or level of physical activity and glioma risk. Additionally, we could not confirm a protective effect of allergic conditions on glioma risk.

### Socio-economic and life-style factors

A higher level of education was associated with an increased risk of glioma in our cohort, although this association was not consistent. We believe that education itself does not increase glioma risk, but may act as a proxy for a higher detection rate. In Norway, healthcare is universally accessible and free of charge, regardless of income or social class. However, the proportion of highly educated individuals is greater in larger cities, where access to advanced diagnostics and specialist care is generally easier. This may contribute to variations in glioma detection rates among individuals with different educational levels.

Although we did not find any association between glioma risk and alcohol consumption, the literature presents mixed results. Cote et al. observed a negative association between alcohol consumption and glioma risk in an analysis of three pooled cohort studies comprising 554 incident glioma cases (19). In the NIH-AARP Diet and Health study by Braganza et al., alcohol consumption was associated with a decreased risk of glioma for both women and men who consumed more than two drinks per day (HR 0.67; 95% CI 0.51-0.90), with evidence of a dose-response relationship (20). In a recent meta-analysis by Shu et al. published in 2022, low to moderate alcohol consumption versus non-drinking was associated with a reduced risk of glioma (RR 0.87; 95% CI 0.78-0.97) (21). However, a previous meta-analysis by Qi et al. did not detect an association between glioma risk and alcohol consumption (RR 0.96; 95% CI 0.89-1.04) (22).

In our study, smoking was not associated with glioma risk, which aligns with the majority of existing literature. Shao et al. conducted a meta-analysis of 19 case-control and 6 cohort studies and found no association with “ever smoking” (RR 0.98; 95% CI 0.92-1.05) overall, or in subgroup analyses for dose-response or age at smoking onset (23). This finding is consistent with another meta-analysis (24). However, a large Chinese case-control study, including 4,556 glioma cases diagnosed using ICD-9 codes, found an increased risk of glioma mortality in smokers overall (OR 1.11; 95% CI 1.03-1.21), with a further increased risk for those smoking ≥20 cigarettes daily for ≥30 years (OR 1.53; 95% CI 1.34-1.74) (25).

### Metabolic factors

Our study generally does not indicate an association between metabolic factors and glioma risk. However, we observed a decreased risk of glioma in men, but not in women, with higher levels of LDL. This association persisted in the multivariable analyses. The role of blood lipids in the pathogenesis of glioma remains unclear. Cote et al. published data from the UK Biobank assessing total cholesterol, HDL, LDL, and triglycerides in relation to glioma risk (26). Overall, neither total cholesterol nor blood lipid subgroups were associated with glioma risk. However, in subgroups excluding the first four years of follow-up, total cholesterol was associated with a significantly higher risk of glioma in men (HR 2.26; 95% CI 1.32-3.89), but not in women (HR 1.28; 95% CI 0.61-2.68) (26). An association with LDL, calculated using the Friedewald equation, was not detected.

The large pooled cohort study by Edlinger et al. reported the risk of glioma in relation to metabolic factors (27). In this study, high levels of triglycerides were associated with an increased risk of high-grade glioma, which remained significant after adjusting for other metabolic factors (HR 1.35 per unit; 95% CI 1.05-1.72). Furthermore, the authors reported that increased diastolic, but not systolic blood pressure, was related to an increased risk of high-grade glioma (HR 1.23 per unit; 95% CI 1.01-1.50) (27).

In a matched case-control analysis of women and men, Seliger et al. found an association between glioma risk and diabetes mellitus (HR 0.81; 95% CI 0.67-0.97), but not with overweight or obesity (28). However, no other metabolic factors were investigated in this study.

In summary, few studies have examined metabolic risk factors in relation to glioma, and the results are inconsistent. Our finding of a negative relationship between glioma risk in men and increasing LDL levels needs further confirmation and should be interpreted with caution.

### Allergic condition

The presence of allergic conditions has been considered a potentially strong protective factor against glioma development. However, our study did not confirm an association between allergic conditions and glioma risk. This contrasts with earlier meta-analyses by Chen et al. and Zhao et al., which reported a decreased risk of glioma in individuals with any allergic condition (OR 0.60; 95% CI 0.52-0.69 and OR 0.78; 95% CI 0.73-0.83, respectively) (14, 15). Additionally, an international case-control study based on INTERPHONE data found an inverse association between glioma and asthma, hay fever, eczema, or any allergy (29).

Conversely, more recent studies, including ours, raise doubts about the protective effect of allergic conditions on glioma risk. For instance, Kaur et al. found conflicting results in a population-based case-control study, with a negative association between glioma risk and asthma in MRI controls (OR 0.5; 95% CI 0.3-0.9), but no association in community controls (30). Anssar et al. reported no association between glioma risk and any allergy, asthma, or hay fever in a large UK case-control study (31). Cahoon et al. observed a significant decrease in primary brain cancer risk among US veterans with long-term allergies, but this finding was inconsistent across subgroups (32). Hemminki et al. found no association between hay fever/allergic rhinitis and glioma risk in a Swedish cohort (33). In contrast, the Taiwan NHIRD study reported an increased risk of brain cancer in subjects with atopic dermatitis or asthma (34).

Our results align with the negative findings of more recent studies, indicating that the protective effect of allergic conditions on glioma risk is not definitive and requires further investigation.

### Study limitations

Achieving sufficient statistical power is challenging in prospective population-based cohort studies investigating low-incidence pathologies. This is evident in our study’s analysis of height and glioma risk in men, which did not find a significant association (HR for highest quartile 1.45; 95% CI 0.94-2.22 and HR per category 1.10; 95% CI 0.96-1.25). However, these effect sizes are comparable to a larger Norwegian cohort study that did find a significant association (HR for highest category 1.43; 95% CI 1.26-1.61 and HR per category 1.21; 95% CI 1.14-1.28) (35). Thus, the lack of significance in our results may be due to insufficient power.

Glioma cases in our study were intracranially located and diagnosed histopathologically, but we could not differentiate them by the latest WHO classification of central nervous system tumors, 5th edition. Gliomas are a heterogeneous group, and it is unclear if they correspond equally to potential risk factors (36). We performed additional analyses for glioblastoma, the largest glioma subgroup, yielding similar but less statistically powerful results (data not shown).

Significant proportions of missing data for some variables (as indicated in the tables) may weaken the validity and statistical power of our findings and introduce bias. Additionally, our study’s external validity is limited as it is based on a Northern-European population, and tumor incidences and potential risk factors may differ in other populations (37, 38).

Factors, such as smoking, alcohol consumption, and metabolic syndrome are closely linked to socio-economic status, which can introduce detection bias in glioma studies and may partly explain inconsistencies between findings. While not a perfect solution, we aimed to minimize this potential bias by using education level as a proxy for socio-economic status and by conducting the study within a universal healthcare system, where access to medical services is independent of socio-economic background.

### Study strengths

The strengths of this study include the prospective follow-up of a population-based cohort of women and men across a wide age range, with an extended follow-up period. The database offers a comprehensive range of variables, enabling detailed analysis of lifestyle, reproductive, and metabolic factors. Measurements of height, weight, and blood pressure were standardized, and blood tests for lipids and glucose included fasting information. Reliable and complete follow-up was ensured through linkage to the National Cancer Registry of Norway and the Norwegian Tax Administration (39).

## Conclusion

This comprehensive prospective cohort study found no strong evidence for modifiable risk factors associated with glioma risk. It also challenges the previously reported 30-40% risk reduction for glioma associated with allergic conditions. Our findings highlight the complexities of glioma risk assessment and suggest directions for further investigation. While we found no strong associations between modifiable lifestyle or metabolic factors and glioma risk, our results underscore the need for nuanced research that considers population-specific variables and advanced diagnostics. The observed link between LDL levels and glioma risk in men, for example, warrants further exploration of lipid metabolism’s role in glioma. New insights could ultimately refine glioma risk profiles, leading to targeted screening in high-risk populations or the development of metabolic or biomarker-based early detection. Our findings emphasize the importance of socio-demographic factors in assessing glioma risk across different populations. While not offering conclusive risk factors, this study provides a foundation for future research into glioma etiology and risk stratification.

## Data Availability

Publicly available datasets were analyzed in this study. The data that support the findings of this study are available from the Cancer Registry of Norway and the Norwegian Institute of Public Health (https://www.fhi.no/en). Restrictions apply to the availability of these data, which were used under license for this study. Requests to access these datasets should be directed to folkehelseinstituttet@fhi.no.
